# The last bastions of guinea-worm disease

**DOI:** 10.2471/BLT.14.021214

**Published:** 2014-12-01

## Abstract

Conflict and a new disease pattern are hampering efforts to eradicate guinea-worm disease in the last four endemic countries. Julius Cavendish reports.

A terrible itchiness is the first symptom of guinea-worm disease or dracunculiasis. Next, explains Adama Guindo, a village chief in eastern Mali, an agonizing blister appears on an ankle or foot. “You don’t see the worm to begin with,” he says. When the creature’s head emerges several days later, the pain is unbearable. Nausea, vomiting, diarrhoea and dizziness follow.

“You can’t work or sleep, and if you can walk at all – it’s with a crutch,” he says. Extracting the worm takes on average between 30 and 60 days, as it must be done manually, by making an incision and twisting the worm slowing out around a stick. Secondary infection from the wound can lead to tetanus, permanent disability or septicaemia, which can be fatal.

“You can’t work or sleep, and if you can walk at all – it’s with a crutch.”

Adama Guindo

Guindo’s home village of Télé, at the foot of the Bandiagara escarpment, a sandstone massif close to the border with Burkina Faso, is one of thousands of communities in Mali that were once ravaged by guinea-worm disease. “We had guinea worm so badly here that women from other villages were frightened of marrying our men,” recalls Ata Lougue.

Only 30 years ago, a large proportion of the population was stricken with this debilitating disease and the granaries were empty, says Professor Ogobara Doumbo, a parasitologist at the University of Mali, who grew up in the area.

Today guinea-worm disease stands on the brink of eradication – the goal is to wipe it out globally by December 2015 – a year from now. Although the eradication goal was revised after an earlier goal to stop human transmission of the disease globally by 2009 was missed, few public health campaigns have made such rapid progress.

In the mid-1980s, 3.5 million new cases were estimated to occur annually, but in 2013, as a result of the intensive efforts to halt transmission, only 148 cases were reported – a reduction of more than 99% since 1989 – and the disease has been confined to four countries: Chad, Ethiopia, Mali and South Sudan.

This year, the pattern has been similar. From January to September, 101 cases have been reported, with 11 of those in Chad, two in Ethiopia, 19 in Mali and 69 in South Sudan.

So far, the World Health Organization (WHO) has certified 197 countries, areas and territories, including 185 WHO Member States, as being free of the disease.

Efforts to wipe out guinea-worm disease date back to the years after smallpox was declared the first disease to be eradicated in 1979.

In May 1981, the Interagency Steering Committee for Cooperative Action for the International Drinking Water Supply and Sanitation Decade (1981–1990) proposed the eradication of guinea-worm disease as an indicator of success for the 10-year clean water campaign.

In the same year, WHO's decision-making body, the World Health Assembly (WHA), adopted resolution WHA 34.25 reinforcing the idea that the International Drinking Water Supply and Sanitation Decade presented an opportunity to wipe out the disease. As a result, WHO and the United States Centers for Disease Control and Prevention developed a strategy and a set of technical guidelines for a global eradication campaign.

To give it a final push in 2011, the WHA called on the countries where the disease was still endemic to step up efforts to stop transmission and strengthen nation-wide surveillance to achieve their goal.

Although the disease is still endemic in Mali, some parts of the country have been transformed by the global eradication campaign. In Télé, children stood giggling shyly when asked about the disease that once ravaged their parents’ communities. “They are too young to know what guinea worm is,” says Lougue, explaining that it has been years since anyone in Télé contracted the disease. In those days, people blamed sorcerers for poisoning their wells – they knew that people got infected from drinking water, but did not know how, she says.

**Figure:**
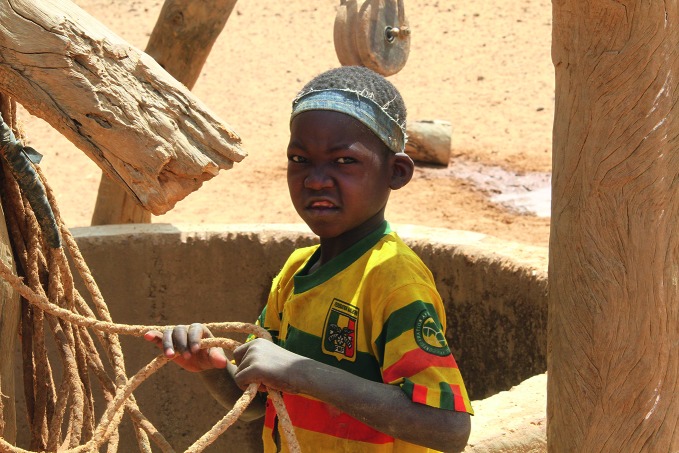
A child in Télé, eastern Mali, hauling water from a deep bore well

Julius Cavendish

A key part of efforts to eradicate the disease has been educating people in the communities affected on how the disease is transmitted. Guinea-worm disease is transmitted via contaminated water, which means that the disease strikes hardest at communities without well pumps, water filters or wells protected by walls: the most impoverished, in other words.

The guinea-worm parasites generally emerge from their victims’ flesh at the same time each year – in Mali’s case during the summer harvest season. The disease traditionally strips communities of labourers at a time when they are most needed. Crops rot in fields. Granaries – buildings of spiritual as well as practical importance, with doors traditionally covered in carvings of sun lizards, crocodiles and ancestor figures – stand empty.

Children with guinea-worm disease are absent from school, while mothers with the disease struggle to look after their children.

Wherever guinea-worm disease strikes, malnutrition and increased poverty follow in its wake. It creates “major barriers to social and economic progress in dozens of countries”, the Carter Center’s Kelly Callahan told a Ted Talks audience last year. “Guinea-worm disease is not a symptom of poverty, but a cause of poverty … So eradicating guinea-worm contributes to ending poverty,” she said. 

In the 1970s, the devastation wrought by guinea-worm disease caught the eye of French parasitologist Philippe Ranque, who began mapping the incidence of guinea-worm disease in Mali. In 1976, Ranque founded the country’s first parasitology department, teaching a cadre of young doctors about the disease’s epidemiology and dispatching them to the field to collect data, and later joined WHO to head the dracunculiasis eradication programme in the 1990s.

When the Carter Center started working on guinea-worm disease eradication in 1986, in partnership with WHO, the United Nations Children’s Fund and health ministries in the then 20 affected countries, these young doctors jumped at their chance.

Guinea-worm disease, they hoped, would become the second human disease in history to be eradicated; and, unlike smallpox, they would do it without administering a single vaccine.

Doumbo, who was one of them, recalls: “Epidemiologically we put everything together to show that it could be eradicated by just changing people’s behaviour.”

The best and cheapest way to do this, he says, is by teaching people to filter their drinking water, to use larvicide to kill water fleas that carry guinea-worm larvae, and to prevent transmission – by keeping anyone who is infected from wading into the drinking water sources, where worms embedded in them can release tens of thousands of larvae into the water to infect new victims.

“You don’t need drugs or a vaccine,” says Doumbo, “just a few simple tools and an effective outreach campaign.”

“You don’t need drugs or a vaccine, just a few simple tools and an effective outreach campaign.”

Ogobara Doumbo

Not only is guinea-worm disease relatively easy to control, in theory, but the benefits of eradication far outweigh the costs. According to a 1997 World Bank study, the economic rate of return on the investment in guinea-worm disease eradication will be about 29% per year once the disease is eradicated – a rate that is based on very conservative estimates of the average amount of time infected workers are unable to work.

So removing guinea-worm disease translates into hundreds of thousands of communities better able to work their fields, send their children to school and escape the cycle of poverty and disease.

But while the techniques for controlling the disease are simple, the last few cases are proving to be the hardest to detect and contain. In Chad, dogs were found to be infected with the same worms that affect humans between 2012 and 2014. So, if infected dogs enter water sources, they too can infect humans – a situation that requires additional containment measures.

**Figure:**
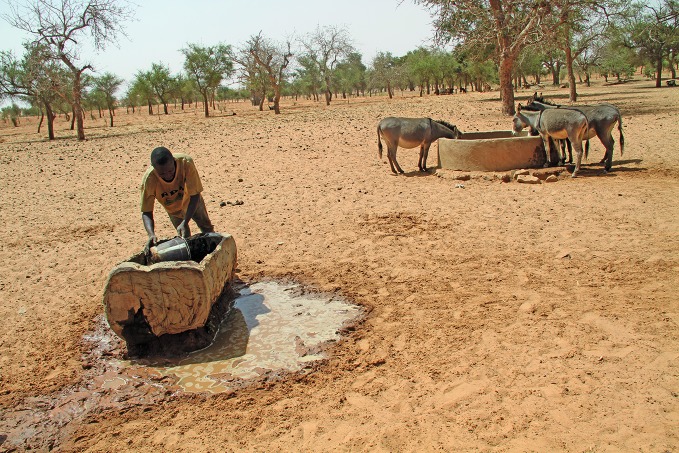
Communal water sources in Télé, eastern Mali

Julius Cavendish

Efforts to wipe out the disease in South Sudan and northern Mali have been hampered by recent conflicts in these countries.

In 2012, for example, Mali reported only seven cases in the whole country – a record low. But since the conflict in northern Mali that year, this rose to 11 in 2013 – 10 of which were in the northern areas affected by the conflict – and from January to September this year, the country’s 19 reported cases were in the northern regions of Gao and Timbuktu.

“After the conflict in 2012, we couldn’t reach the northern regions,” says Dr Gabriel Guindo, the Malian health ministry official in charge of the national dracunculiasis eradication programme.

“This meant that people were able to contaminate water supplies and infect others,” he says, adding that since the French military intervention, government health workers have been slowly returning to Mali’s northern regions to do the necessary prevention work.

“If we have access to all the regions without any problem,” Guindo reckons, then “by 2016 the problem should be solved.”

